# Pannexin-1 is present in a subpopulation of bovine milk–derived small extracellular vesicles

**DOI:** 10.1007/s00441-026-04044-x

**Published:** 2026-01-27

**Authors:** Md Ruhul Amin, Spencer R. Marsh, Claire Beard, Laura Beth Payne, Amanda Charest, Randy F. Stout, Robert G. Gourdie

**Affiliations:** 1 Center for Vascular and Heart Research, Fralin Biomedical Research Institute at Virginia Tech Carilion, Roanoke, VA 24016 USA; 2 Translational Biology, Medicine, and Health Graduate Program, Virginia Tech, Roanoke, VA 24016 USA; 3 Department of Biomedical Sciences, College of Osteopathic Medicine, New York Institute of Technology, Old Westbury, NY 11568 USA; 4 Virginia Tech Carilion School of Medicine, Roanoke, VA 24016 USA; 5 Department of Biomedical Engineering and Mechanics, Virginia Polytechnic Institute and State University, Blacksburg, VA 24060 USA

**Keywords:** Pannexin-1, Milk-derived small extracellular vesicles, Flow cytometry-based vesicle detection, Single molecule localization microscopy, Vesicle protein cargo

## Abstract

Pannexin-1 (Panx1) is a large-pore membrane channel protein implicated in diverse physiological and pathological processes, yet its presence in extracellular vesicles has not been fully characterized. In this study, we investigated small extracellular vesicles derived from bovine milk to determine whether they contain Panx1. Using multiple complementary approaches, we show that Panx1 is present in a distinct subpopulation of milk-derived vesicles. Panx1 immunoreactivity was detected by Western blotting with antibodies targeting both the C-terminal and extracellular loop regions, and single-molecule localization microscopy revealed that a subpopulation of CD9-positive mEVs expresses Panx1. Consistently, flow cytometry–based analysis indicated that approximately half of the vesicle population was Panx1 positive. Together, these findings provide the first validated evidence that Panx1 is incorporated into bovine milk–derived small extracellular vesicles, expanding the repertoire of membrane channel proteins associated with extracellular vesicles and establishing a foundation for future studies on the functional role of Panx1 in vesicle biology and vesicle-mediated signaling.

**Supplementary information:**

The online version contains supplementary material available at 10.1007/s00441-026-04044-x.

## Introduction

Small extracellular vesicles (EVs) with a lipid bilayer, and a diameter of less than 200 nm, are secreted by most cells and are vital in intercellular communication as they contain various proteins, lipids, DNA, miRNAs, and other ncRNAs (Marsh et al. 2025). They are abundant in many biological fluids, including milk, and milk-derived small extracellular vesicles (mEVs) have attracted attention for their stability, bioavailability, and potential roles in neonatal development and immune modulation (Manca et al. 2018; Marsh et al. 2025; Rashidi et al. 2022). Moreover, in recent times, mEVs have emerged as promising nanocarriers for drug delivery due to their natural abundance, safety profile, stability, ability to be orally administered, and high bioavailability once ingested (Marsh et al. 2025; Schifano et al. 2025). Additionally, mEVs invoke minimal immune responses, demonstrate reduced clearance by first-pass metabolism, and an ability to cross biological barriers like the blood–brain barrier—all features that enhance their therapeutic potential (Somiya et al. 2018; Zempleni 2017).

Direct intercellular communication can occur between neighboring cells via gap junctions or indirectly across larger distances by releasing soluble substances and small EVs into the surrounding environment. Recent studies showed evidence that these processes might overlap by reporting that small EVs contain subunit gap junction proteins, Connexins (Gemel et al. 2019; Martins-Marques et al. 2019). Soares et al. first reported evidence for a role of Connexin-43 (Cx43) in EV-mediated communication (Soares et al. 2015). In our prior work, we reported that Cx43 is also present in a subpopulation of mEVs (Amin et al. 2024). This finding led us to investigate whether Pannexin-1 (Panx1), another membrane channel protein similar to Cx43, is also present in mEVs.

Since pannexins were first described more than two decades ago (Panchina et al. 2000), research has largely focused on their roles in the vasculature and in organs characterized by high vascular density, such as the heart, lungs, and brain (O’Donnell et al. 2025). Within this family, pannexin-1 has emerged as the most extensively studied isoform, reflecting its widespread expression across blood and lymphatic vessels and growing evidence that altered Panx1 activity contributes to vascular dysfunction and disease (O’Donnell et al. 2025). Panx1 is a large-pore forming glycoprotein known primarily for functioning as an ATP-release channel in the plasma membrane, regulating purinergic signaling, inflammation, and cell death pathways (Chekeni et al. 2010; Penuela et al. 2014; Yang et al. 2020).

While the role of Panx1 is well-established in plasma membranes, its association with extracellular vesicles is an emerging area of study. Importantly, in a recent study, Panx1 activity has been linked to the secretion of exosomal miRNAs in hepatitis C virus–infected hepatocytes, suggesting a regulatory link between this channel and the release of vesicular cargo (Kim et al. 2021). Despite evidence that Panx1 influences extracellular vesicle secretion, prior literature lacks evidence of PANX1 as a well-validated cargo protein within small EVs/exosomes, especially from noncell culture fluids like milk. Proteomic analyses of small extracellular vesicles isolated from cultured cell lines, including gastric and ovarian cancer cells, have reported the presence of Panx1 (PANX1; gene ID 24145) in mass spectrometry datasets. However, these observations were limited to peptide-level identifications and were not accompanied by validation using orthogonal approaches. To our knowledge, no previous studies have comprehensively examined the localization or relative abundance of Panx1 in small extracellular vesicles derived from cell culture systems or biological fluids, including milk.

The aim of this study is to determine whether pannexin-1 is present in small extracellular vesicles derived from bovine milk and to define its distribution among vesicle subpopulations. Here, we report, for the first time, that PANX1 is present in a subpopulation of small EVs derived from bovine milk using multiple complementary approaches. This finding expands the known protein repertoire of mEVs and establishes a critical foundation for investigating its functional contribution to exosomal stability, cargo transport, and overall physiological significance.

## Materials and methods

### Isolation of small EVs from bovine milk

EV isolation protocol used was based on our previously reported method by Marsh and co-worker (Marsh et al. 2021). The key step of this method was the chemical solubilization of casein micellar structures by divalent cation chelation with 30 mM EDTA at 37 °C for 1 h. In brief, raw bovine milk at 4 °C was obtained from Homestead Creamery of Wirtz, VA after morning milking. Subsequent protocol steps, except the 1-h 37°C EDTA treatment, were performed at 4 °C. Milk was transferred to sterile polypropylene tubes (Thermo Scientific, 75,007,585) and centrifuged at 5000 rcf (Sorval Legend X1R centrifuge with Sorval TX-400 75,003,629 rotor) for 30 min. Fat was removed by whisking the creamy upper layer of supernatant (SN) with filter paper and decanting the remaining SN from the pellet. The centrifugation, whisking, and SN decanting steps were then repeated. The resultant SN was transferred to 250-ml centrifuge tubes (Nalgene) and centrifuged at 14,500 rcf (Beckmann Coulter Avanti, J-26 XP centrifuge with JLA 16.25 rotor) for 60 min. SN was then decanted from the pellet, and this SN was then centrifuged at 22,600 rcf for 60 min four consecutive times, with the pellet being discarded after each spin. This solution was then filtered through 0.45- and 0.22-μm filters (MilliporeSigma) in sequence and then treated with 30 mM EDTA at 37 °C for 60 min. The EDTA-treated filtrate was then subject to cross-flow filtration using a Repligen Krosflo Tangential Flow Filtration (TFF) system with a 500-kDa MidiKros TFF Filter (Repligen). Once the TFF solution reached ~ 20% of starting volume, the EV-containing solution was diafiltered in sterile, degassed 10 × volume HEPES buffer (100 mM NaCl, 20 mM HEPES, 4 mM KCl, pH 7.5), aliquoted in 1-ml volumes, and stored at − 80 °C. Following thawing, solutions containing EV concentrates were further processed by purification on an IZON qEV original 70-nm sepharose column (IZON, 1,006,881) and collected manually in a 96-well plate to allow uniform fraction tracking and precise downstream pooling based on protein and particle measurements. Based on protein concentration, peak EV fractions (Fractions 7–9) were pooled and analyzed, including by Nanodrop, Nanosight, and spectrophotometry. For storage, purified mEVs were lyophilized and stored as a freeze-dried powder as we described previously (Dogan et al. 2025).

### Gel electrophoresis and Western blot (WB)

Gel electrophoresis and WB analysis was performed as previously described (Marsh et al. 2021). Samples were separated by sodium dodecyl sulfate polyacrylamide gel electrophoresis (SDS-PAGE) and transferred to a PVDF (MilliporeSigma, IPFL00010) membrane. Membranes were blocked in EveryBlot Blocking Buffer (Bio-Rad Laboratories, cat. # 12,010,020) for 5 min at room temperature. Overnight primary antibody incubation was performed, and primary antibodies were diluted in the blocking buffer as follows: CD81 (Cell Signaling Technology, 56039S, 1:1000), CD9 (Novus Biologicals, NB500-494, 1:1000), Calnexin (MilliporeSigma, AB2301, 1:5000), TSG101 (Bethyl Laboratories Inc. A303-506A, 1:5000), Syntenin (LSBio, LS-C40484, 1:1000), Casein (Abcam, Ab166596, 1:2000), Panx1 extracellular-loop (LSBio, LS-C30513, 1:1000), and antibodies against the Panx1 CT (MilliporeSigma, HPA016930, 1:1000). Following washing, the membrane was incubated for 1 h at room temperature with secondary antibodies diluted 1:20,000 for mouse (Jackson ImmunoResearch, 715–035–150) and 1:20,000 for rabbit (Southern Biotechnology, 4050–05) in blocking buffer. Proteins of interest were visualized by chemiluminescence using a Bio-Rad ChemiDoc MP imager. In total, three samples were analyzed for WB with each antibody.

### Flow cytometry–based vesicle detection

To stain mEVs with dye, samples were loaded with cell tracker deep red (CTDR) (Invitrogen, C34565) at 100 nM for 2 h at 37° C, then centrifuged at 16,873 × g for 1 h at 4 °C to remove unincorporated dye. Supernatants were discarded, and pellets were resuspended in HEPES buffer. For detection of Panx1 in mEVs, pellets of 250 μl of mEVs were resuspended in 250 μl of PEB buffer (PBS, 5 mM EDTA, and 0.5% BSA) and were blocked with 100 μg/ml of rabbit IgG for 2 h at RT. After blocking, the samples were washed with 1 ml of PEB buffer. The samples were then incubated overnight with Panx1 extracellular-loop (LSBio, LS-C30513, 0.5 μg/ml) directly conjugated to Alexa-488 dye, using the FlexAble antibody labeling kit (Proteintech). After incubation, the labeled mEV samples were washed twice with 1 ml of PEB buffer and resuspended in 200 μl of PEB buffer (PBS, 5 mM EDTA, and 0.5% BSA), followed by another wash using a protein desalting spin column (Thermo # 89,849) to remove nonbound antibody. For flow cytometry–based vesicle detection, samples were collected on a BD Biosciences FACS Fusion Flow Cytometer. EVs were displayed on an FSC-A and SSC-A dot plot using log scale. A gate was drawn around the EVs, and a subsequent plot of FSC-H (log) vs FSC-W (lin) was displayed and gated to remove aggregate events. Acquisition was set to record 10,000 singlet events per sample. Alexa 488 was detected with a 488-nm laser excitation and a 530/30 bandpass filter. After acquisition, the same gating methods were employed to analyze the samples using FlowJo software VX. In total, seven samples were analyzed by this method (four for single stain and three for double stain).

### Nanoparticle tracking analysis (NTA)

NTA was performed on a NanoSight NS300 (Malvern Panalytical) at 20 °C. EV concentrates obtained post-SEC were diluted 1:10 in HEPES buffer, sonicated in a Branson 2510 bath sonicator for 30 s to reduce vesicle aggregation and ensure a homogeneous suspension. EV samples were then diluted (1:1000 to 1:10,000 depending on sample) and loaded into the NanoSight low-volume flow cell. Each sample was analyzed using a 405-nm laser with 5 consecutive 1-min recordings at a constant flow of 10, as per Malvern software. Videos were analyzed using NTA software (Version 3.4) and nanoparticle counts and dimensions generated as we have previously reported (Marsh et al. 2021). In total, NTA of ten samples was performed to validate the results.

### Transmission electron microscopy (TEM)

Prior to sample loading, formvar-coated copper grids (Electron Microscopy Services, FCF200-CU-50) were glow discharged using a Pelco Easy Glo (Ted Pella). For each following step, solution volume was 10 μl, and solution was removed by gently wicking with a small square of Whatman filter paper #1. Grids were coated with 0.01% lysine for 60 s, then rinsed twice in 10 μl ddi water from a Millipore unit. Samples were loaded onto lysine-coated grids for 10 min, then counterstained with uranyl-ess (Electron Microscopy Services, 22,409–20). Samples were stored in a standard grid box at room temperature prior to TEM imaging. In total, 10 grids were prepared and imaged for this study.

### Single-molecule localization microscopy

Single-molecule localization microscopy (SMLM) was performed in collaboration with the New York Institute of Technology (NYIT) and Nanometrix. The EV Profiler Kit (Oxford Nanoimaging) containing antibodies against the tetraspanins—CD9, CD63, and CD81—was used according to the manufacturer’s instructions. Briefly, 2.5 µl of sample was gently mixed with 3.5 µl blocking buffer and allowed to incubate for 5 min prior to the addition of fluorophore-conjugated antibodies. In addition to antibodies provided in the kit, Panx1 antibody (LSBio, LS-C30513) conjugated to Alexa Fluor 488 was also used to detect Panx1 in mEVs. When necessary, PBS wash buffer was used to reach the final sample volume of 9 µl. Samples were allowed to incubate overnight at 4 °C protected from light. The following day, samples, buffers, and the chip were brought to room temperature. Immediately after surface preparation of the chip, the samples were mixed with the EV capture solution and loaded into the lanes on the chip. The chip with samples was incubated for 15 min protected from light, whereafter wash and fixation steps were performed. Immediately prior to imaging, freshly mixed Bcubed buffer was added to the lanes. Imaging was then performed, and Nanometrix software was used for analysis and data output.

## Results

### Isolation and characterization of bovine milk–derived small extracellular vesicles

**Fig. 1:**
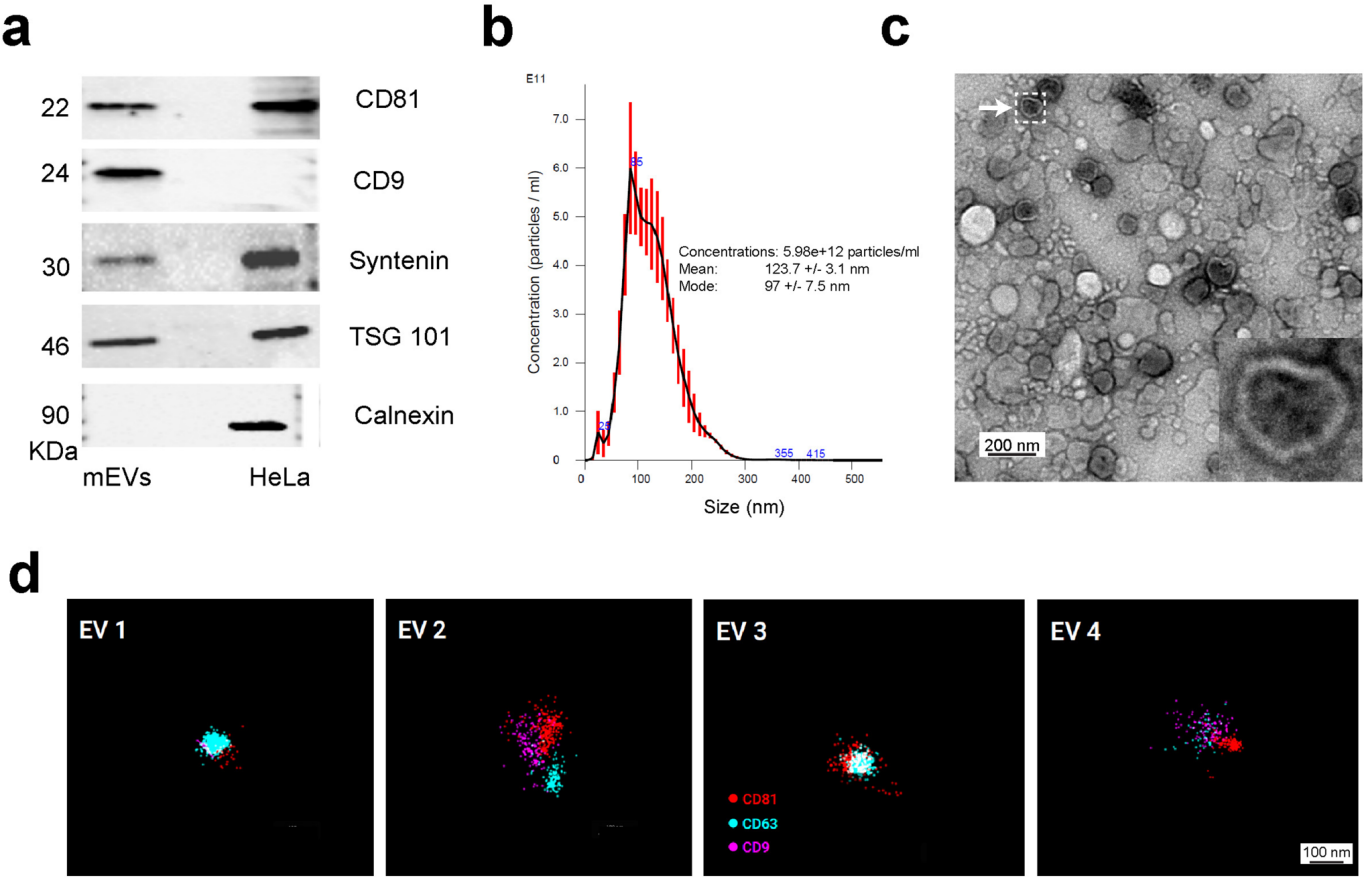
Milk-derived small extracellular vesicles (mEVs) characterization. **a** Western blotting of mEV isolates for CD81, CD9, Syntenin, TSG-101, and Calnexin. Lysates from HeLa cells are included as a control. **b** Nanoparticle tracking analysis (NTA) of an mEV isolate indicating > 5 × 10^12^ mEVs per ml, with an average nanoparticle size of 123.7 nm, consistent with small EVs. **c** Negative stain transmission electron microscopy (TEM) indicating dense accumulations of mEVs generated by the isolation protocol. A five times zoom-in image of a single mEV in the inset shows that isolated mEVs maintained their characteristic spherical morphology and intact lipid bilayers. **d** Single-molecule localization microscopy (SMLM) images of an isolated mEV tagged for CD81 (red), CD9 (pink), and CD63 (teal)

We isolated small extracellular vesicles from unpasteurized bovine milk using our previously published method (Marsh et al. 2021), which uses EDTA to free mEVs from milk protein complexes, including casein micelles. We used tangential flow filtration (TFF) to harvest disassociated mEVs, thereby eliminating the ultracentrifugation (UC) step commonly utilized in most extracellular vesicle (EV) isolation protocols. TFF followed by Sepharose column (SEC) fractionation leads to improvements in mEV yield, retaining mEV integrity and enabling production of mEVs at scale (Marsh et al. 2021). After isolation, we characterized the mEVs by Western blotting (WB), nanoparticle tracking analysis (NTA), and transmission electron microscopy (TEM)—confirming the presence of relatively pure and ultra-structurally definitive mEVs at high density (Fig. 1). Following TFF and size-exclusion chromatography (SEC), transmission electron microscopy (TEM) confirmed that the isolated mEVs maintained their characteristic spherical morphology and intact lipid bilayers (Fig. 1c and Sup. Figure 1). Nanoparticle tracking analysis (NTA) indicated a homogeneous population of vesicles with a mean diameter of 123.7 ± 3.1 nm and a single-peaked size distribution, with particle concentrations consistently exceeding 5 × 10^12^ particles/ml (Fig. 1b). Western blot analysis of the pooled mEV fractions demonstrated enrichment for canonical small EV markers, including CD9, CD81, Syntenin, and TSG-101, while Calnexin—a negative marker for small extracellular vesicles and an endoplasmic reticulum marker—was not detected (Fig. 1a). Casein levels in the pooled EV fraction were also probed by Western blotting to confirm reduction relative to later non-EV containing SEC fractions (Fraction 16, F-16) as shown in supplemental Fig. 2c. Moreover, we performed single-molecule localization microscopy (SMLM) to characterize mEVs at super-resolution. SMLM imaging, performed in collaboration with Nanometrix and the New York Institute of Technology, enabled visualization of individual vesicles and colocalization of surface markers such as CD9, CD63, and CD81 (Fig. 1d).

#### Pannexin-1 is present in milk EV subpopulation

**Fig. 2:**
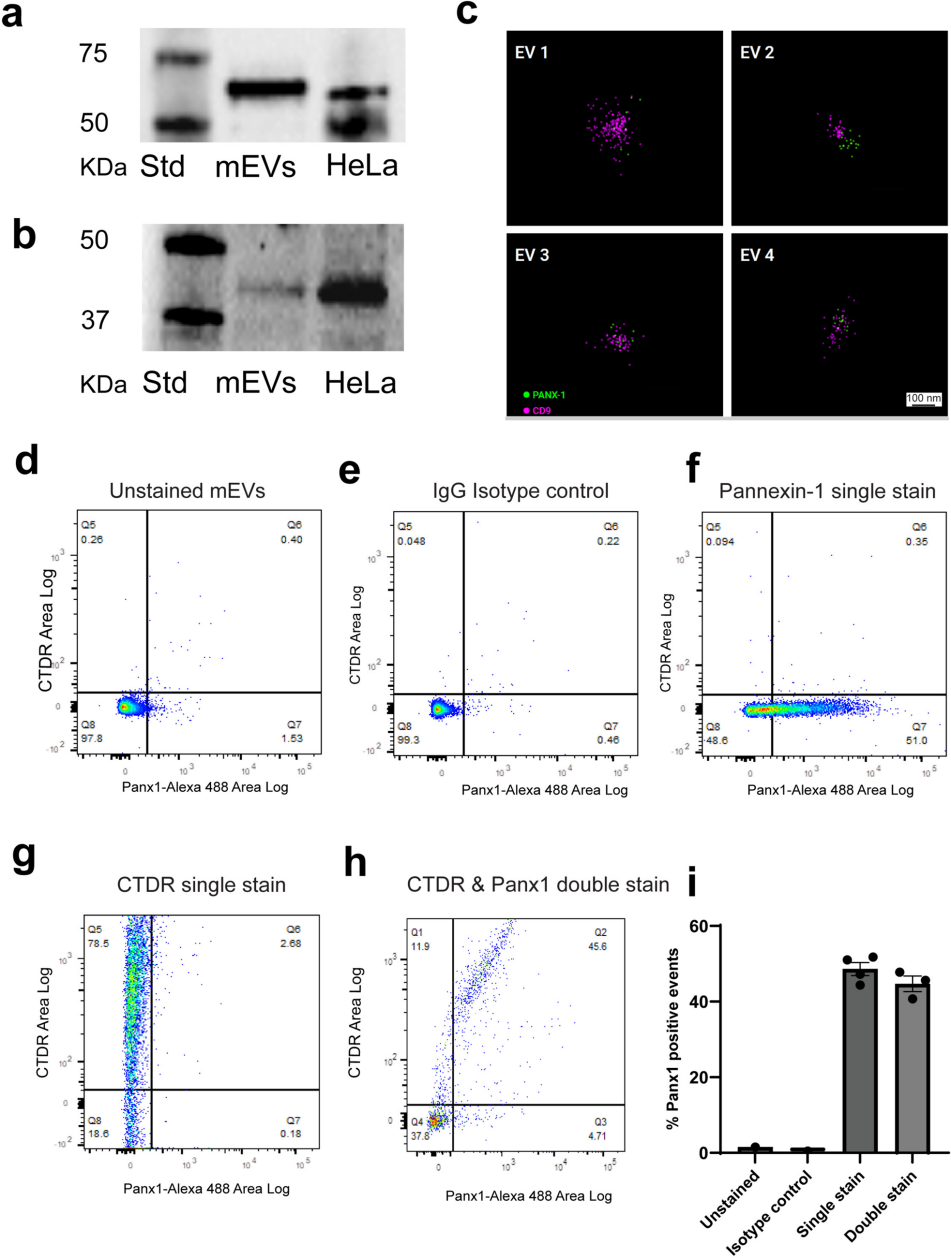
A subpopulation of mEVs containsPannexin-1. **a** Western blots for Panx1 in mEV lysates and control lysates of HeLa cells with Panx1 CT antibody. **b** Western blots for Panx1 in mEV lysates and control lysates of HeLa cells with Panx1 extracellular loop antibody. **c** SMLM imaging of mEVs detected by Panx1 antibody (green) together with CD9 (co-labeling (pink). **d**–**h** Flow cytometry–based detection of mEVs containing Panx1. **d** Samples were unstained, **e** stained with isotype IgG, **f** single stained with Alexa-488-labelled Panx1, **g** single stained with CTDR dye, or **h** simultaneously stained with CTDR and Alexa-488-labelled Panx1. **i** Quantification of flow cytometry-based vesicle detection of mEVs positive for Panx1, as detected by single stain and double stain. *N* = 4 for single stain and *N* = 3 for double stain, error bars = SEM. Percentages of gated populations are shown

To determine whether pannexin-1 (Panx1) is present in mEVs, we employed multiple complementary approaches. Western blotting of mEV lysates revealed clear immunoreactive bands corresponding to Panx1, with migration patterns consistent with HeLa cell lysates used as positive controls, as HeLa cell lines have been reported to express Panx1 (Dvoriantchikova et al. 2006). Two different antibodies with distinct epitopes were used to confirm the result. One directed against the C-terminal region showed a band at 50–55 KDa (Fig. 2a and Sup. Figure 2a) and another against the extracellular loop of Panx1 (aa 231–280) detected a signal at 45–50 KDa (Fig. 2b and Sup. Figure 2b). Notably, the relative MW matched the control HeLa cell lysates and the manufacturers' expected MW for full-length Panx1 in WB for each of the two antibodies, respectively. Single-molecule localization microscopy (SMLM) further confirmed the presence of Panx1 in mEVs. In SMLM, some but not all CD9-positive mEVs displayed Panx1 labeling (Fig. 2c), indicating that Panx1 is incorporated into a defined subpopulation of mEVs. To further investigate the Panx1-positive subpopulation of vesicles, we undertook flow cytometry–based vesicle detection of mEVs. Unstained (Fig. 2d) and isotype IgG-stained mEVs (Fig. 2e) showed minimal background fluorescence and were used to set the fluorescent gates. Vesicle flow cytometry analysis of four replicates showed that, on average, 48.6% of detected vesicles were positive for Panx1 when stained individually, and 44.7% of CTDR-labeled small EVs exhibited Panx1 staining in double-labeling experiments (Fig. 2f–i). Thus, both SMLM and flow cytometry-based vesicle detection indicated the presence of a subpopulation within the broader population of mEVs that was positive for Panx1.

## Discussion

In this study, we provide the first evidence that pannexin-1 (Panx1) is present as a protein cargo in bovine milk-derived small EVs. Using Western blotting with two distinct antibodies, single-molecule localization microscopy and vesicle flow cytometry, we consistently detected Panx1 in a subpopulation of mEVs, with nearly half of small vesicles exhibiting Panx1 staining. This heterogeneity reinforces the concept that mEV populations are mixed and that cargo composition varies among vesicle subtypes. These findings extend the current understanding of mEV composition and suggest that Panx1 is not only a regulator of extracellular vesicle secretion, as previously reported (Phan et al. 2021), but also an integral small EV protein.

Previous studies have implicated Panx1 channels in ATP release, purinergic signaling, and inflammation, as well as in regulating exosome biogenesis and cargo secretion (Chekeni et al. 2010; Kim et al. 2021; Penuela et al. 2014; Phan et al. 2021; Yang et al. 2020). However, no prior proteomic studies, including those from bovine milk, have reported Panx1 as a vesicular component (Samuel et al. 2017; Wang et al. 2020). Our findings therefore highlight a previously unrecognized aspect of Panx1 biology, namely, its incorporation into extracellular vesicles themselves. The presence of Panx1 in small EVs raises intriguing questions about its functional role. Looking forward, it will be valuable to examine Panx1’s presence in EVs derived from other sources to see whether the occurrence of Panx1 in exosomes is a generalizable phenomenon or limited to specific tissues or fluids. Functional assays should test whether Panx1 in small EVs is active (e.g., mediates ATP flux and influences permeability or recipient cell signaling) and whether Panx1-positive small EVs differ in uptake or biological effects versus Panx1-negative small EVs subpopulations.

Milk-derived small EVs are increasingly recognized as bioactive mediators capable of crossing species barriers and exerting systemic effects (Marsh et al. 2025). The detection of Panx1 in mEVs opens new avenues for exploring whether mEV-associated Panx1 contributes to immune regulation, metabolic adaptation, or intercellular communication in the neonate or other consumers of milk. Further work is needed to determine whether Panx1-containing mEVs exhibit distinct biological properties compared to Panx1-negative vesicles and to elucidate whether Panx1 remains functional as a channel protein in the mEV membrane similar to Cx43 (Soares et al. 2015).

In summary, this study identifies Panx1 as a novel cargo of bovine milk-derived exosomes. These findings broaden the molecular landscape of mEVs and raise new hypotheses regarding the functional significance of Panx1 in extracellular vesicle-mediated signaling.

## Supplementary information

Below is the link to the electronic supplementary material.

ESM 1 Full-length, uncropped transmission electron microscopy images of mEVs in different magnification scales (a–c) (PNG 638 KB)

ESM 1 (TIF 11.6 MB)

ESM 2 Original, uncropped blot images, including merged molecular weight markers. a) Western blots for Cx43 in mEV lysates and control lysates of HeLa cells with Panx1 CT antibody. b) Western blots for Cx43 in mEV lysates and control lysates of HeLa cells with Panx1 extracellular loop antibody. c) Western blot for casein in the pooled EV fraction, non-EV containing SEC fractions (Fraction 16, F-16) and control lysates of HeLa cells (PNG 252 KB)

ESM 2 (TIF 11.4 MB)

## Data Availability

Data is provided within the manuscript or supplementary information files.

## References

[CR1] Amin MR, Marsh S, Beard C, Jourdan J, Gourdie R (2024) Abstract Tu005: small extracellular vesicles derived from bovine milk contain an endogenous carboxyl terminal polypeptide of the gap junction protein connexin-43. Circ Res 135(Suppl_1):ATu005–ATu005. 10.1161/res.135.suppl_1.Tu005

[CR2] Chekeni FB, Elliott MR, Sandilos JK, Walk SF, Kinchen JM, Lazarowski ER, Armstrong AJ, Penuela S, Laird DW, Salvesen GS, Isakson BE, Bayliss DA, Ravichandran KS (2010) Pannexin 1 channels mediate ‘find–me’ signal release and membrane permeability during apoptosis. Nature 467(7317):863–867. 10.1038/nature09413 20944749 10.1038/nature09413 PMC3006164

[CR3] Dogan AB, Marsh SR, Tschetter RJ, E. Beard C, Amin MR, Jane Jourdan L, Gourdie RG (2025) Stabilizing milk-derived extracellular vesicles (mEVs) through lyophilization: a novel trehalose and tryptophan formulation for maintaining structure and bioactivity during long-term storage. J Biol Eng 19(1):4. 10.1186/s13036-024-00470-z 39806456 10.1186/s13036-024-00470-z PMC11727230

[CR4] Dvoriantchikova G, Ivanov D, Pestova A, Shestopalov V (2006) Molecular characterization of pannexins in the lens. Mol Vis 12:1417–1426. http://www.molvis.org/molvis/v12/a160/17149368

[CR5] Gemel J, Kilkus J, Dawson G, Beyer EC (2019) Connecting exosomes and connexins. Cancers 11(4):4. 10.3390/cancers11040476 10.3390/cancers11040476 PMC6520873 30987321

[CR6] Kim O-K, Nam D, Hahn YS (2021) The Panx1/P2X4 pathway controls the secretion of miRNA-containing exosomes by HCV-infected hepatocytes. Hepatology (Baltimore, MD) 74(6):3409–3426. 10.1002/hep.32042 34218459 10.1002/hep.32042 PMC8639610

[CR7] Manca S, Upadhyaya B, Mutai E, Desaulniers AT, Cederberg RA, White BR, Zempleni J (2018) Milk exosomes are bioavailable and distinct microRNA cargos have unique tissue distribution patterns. Sci Rep 8(1):1. 10.1038/s41598-018-29780-1 30054561 10.1038/s41598-018-29780-1 PMC6063888

[CR8] Marsh SR, Pridham KJ, Jourdan J, Gourdie RG (2021) Novel protocols for scalable production of high quality purified small extracellular vesicles from bovine milk. Nanotheranostics 5(4):4. 10.7150/ntno.62213 10.7150/ntno.62213 PMC8342262 34367882

[CR9] Marsh SR, Beard CE, Gourdie RG (2025) Milk extracellular vesicles: a burgeoning new presence in nutraceuticals and drug delivery. Bioeng Transl Med 10(3):e10756. 10.1002/btm2.10756 40385535 10.1002/btm2.10756 PMC12079498

[CR10] Martins-Marques T, Ribeiro-Rodrigues T, Batista-Almeida D, Aasen T, Kwak BR, Girao H (2019) Biological functions of connexin43 beyond intercellular communication. Trends Cell Biol 29(10):835–847. 10.1016/j.tcb.2019.07.001 31358412 10.1016/j.tcb.2019.07.001

[CR11] O’Donnell BL, Williams MD, Billaud M, Dunaway LS, Columbus L, Koval M, Isakson BE (2025) Pannexins in the vasculature. Am J Physiol Heart Circ Physiol 329(6):H1449–H1470. 10.1152/ajpheart.00510.2025 41051983 10.1152/ajpheart.00510.2025 PMC12560215

[CR12] Panchina Y, Kelmanson I, Matz M, Lukyanov K, Usman N, Lukyanov S (2000) A ubiquitous family of putative gap junction molecules. Curr Biol 10(13):R473–R474. 10.1016/S0960-9822(00)00576-5 10898987 10.1016/s0960-9822(00)00576-5

[CR13] Penuela S, Simek J, Thompson RJ (2014) Regulation of pannexin channels by post-translational modifications. FEBS Lett 588(8):1411–1415. 10.1016/j.febslet.2014.01.028 24486011 10.1016/j.febslet.2014.01.028

[CR14] Phan TK, Fonseka P, Tixeira R, Pathan M, Ang C-S, Ozkocak DC, Mathivanan S, Poon IKH (2021) Pannexin-1 channel regulates nuclear content packaging into apoptotic bodies and their size. Proteomics 21(13–14):e2000097. 10.1002/pmic.202000097 33661579 10.1002/pmic.202000097

[CR15] Rashidi M, Bijari S, Khazaei AH, Shojaei-Ghahrizjani F, Rezakhani L (2022) The role of milk-derived exosomes in the treatment of diseases. Front Genet 13:1009338. 10.3389/fgene.2022.1009338 36338966 10.3389/fgene.2022.1009338 PMC9634108

[CR16] Samuel M, Chisanga D, Liem M, Keerthikumar S, Anand S, Ang C-S, Adda CG, Versteegen E, Jois M, Mathivanan S (2017) Bovine milk-derived exosomes from colostrum are enriched with proteins implicated in immune response and growth. Sci Rep 7(1):5933. 10.1038/s41598-017-06288-8 28725021 10.1038/s41598-017-06288-8 PMC5517456

[CR17] Schifano E, Vari F, Buccini L, Karimova M, Syman K, Varnadyan D, Uccelletti D, Dinarelli S, Zuccotti M, Alfieri A, Sennato S, Mura F, Rossi M, Dini L, Tacconi S (2025) A novel scalable method for the production of rennet-treated milk-derived extracellular vesicles for improved curcumin oral delivery. J Nanobiotechnology 23(1):656. 10.1186/s12951-025-03724-0 41074180 10.1186/s12951-025-03724-0 PMC12514809

[CR18] Soares AR, Martins-Marques T, Ribeiro-Rodrigues T, Ferreira JV, Catarino S, Pinho MJ, Zuzarte M, Isabel Anjo S, Manadas B, P G Sluijter J, Pereira P, Girao H (2015) Gap junctional protein Cx43 is involved in the communication between extracellular vesicles and mammalian cells. Sci Rep 5:13243. 10.1038/srep13243 26285688 10.1038/srep13243 PMC4541155

[CR19] Somiya M, Yoshioka Y, Ochiya T (2018) Biocompatibility of highly purified bovine milk-derived extracellular vesicles. J Extracell Vesicles 7(1):1440132. 10.1080/20013078.2018.1440132 29511463 10.1080/20013078.2018.1440132 PMC5827637

[CR20] Wang Z, He Z, Liang S, Yang Q, Cheng P, Chen A (2020) Comprehensive proteomic analysis of exosomes derived from human bone marrow, adipose tissue, and umbilical cord mesenchymal stem cells. Stem Cell Res Ther 11(1):511. 10.1186/s13287-020-02032-8 33246507 10.1186/s13287-020-02032-8 PMC7694919

[CR21] Yang Y, Delalio LJ, Best AK, Macal E, Milstein J, Donnelly I, Miller AM, McBride M, Shu X, Koval M, Isakson BE, Johnstone SR (2020) Endothelial pannexin 1 channels control inflammation by regulating intracellular calcium. J Immunol 204(11):2995–3007. 10.4049/jimmunol.1901089 32312847 10.4049/jimmunol.1901089 PMC7336877

[CR22] Zempleni J (2017) Milk exosomes: beyond dietary microRNAs. Genes Nutr 12:12. 10.1186/s12263-017-0562-6 28694874 10.1186/s12263-017-0562-6 PMC5501576

