# Supplementary figures and images for: Pannexin-1 is present in a subpopulation of bovine milk–derived small extracellular vesicles
Source: Cell Tissue Res. 2026 Jan 27;403(2):13. doi: 10.1007/s00441-026-04044-x (PMC12847120; doi:10.1007/s00441-026-04044-x)

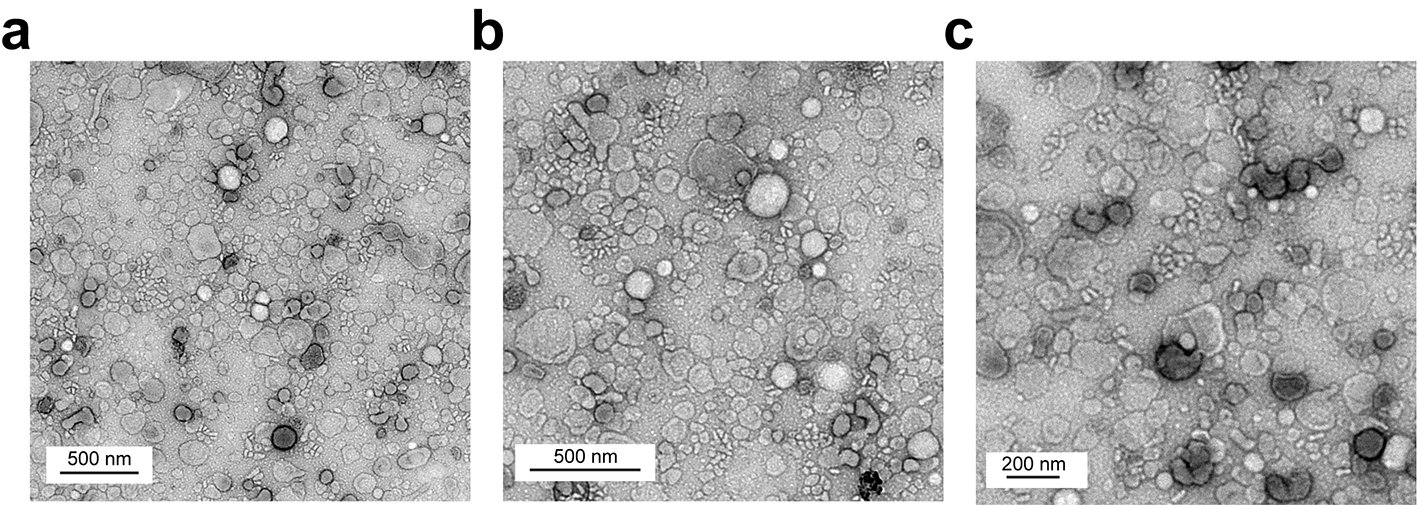

Supplement: Supplementary file 1 — Full-length, uncropped transmission electron microscopy images of mEVs in different magnification scales (a–c) (PNG 638 KB) [file 441_2026_4044_Fig3_ESM.png]

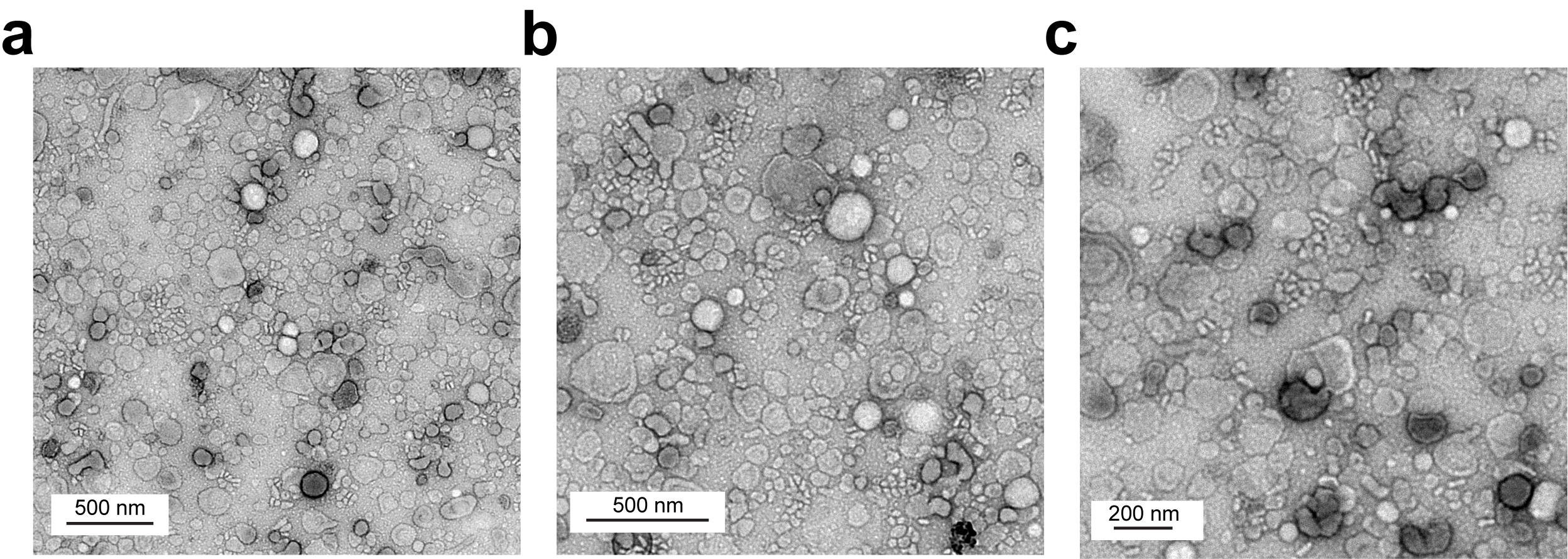

Supplement: Supplementary file 2 — (TIF 11.6 MB) [file 441_2026_4044_MOESM1_ESM.tif]

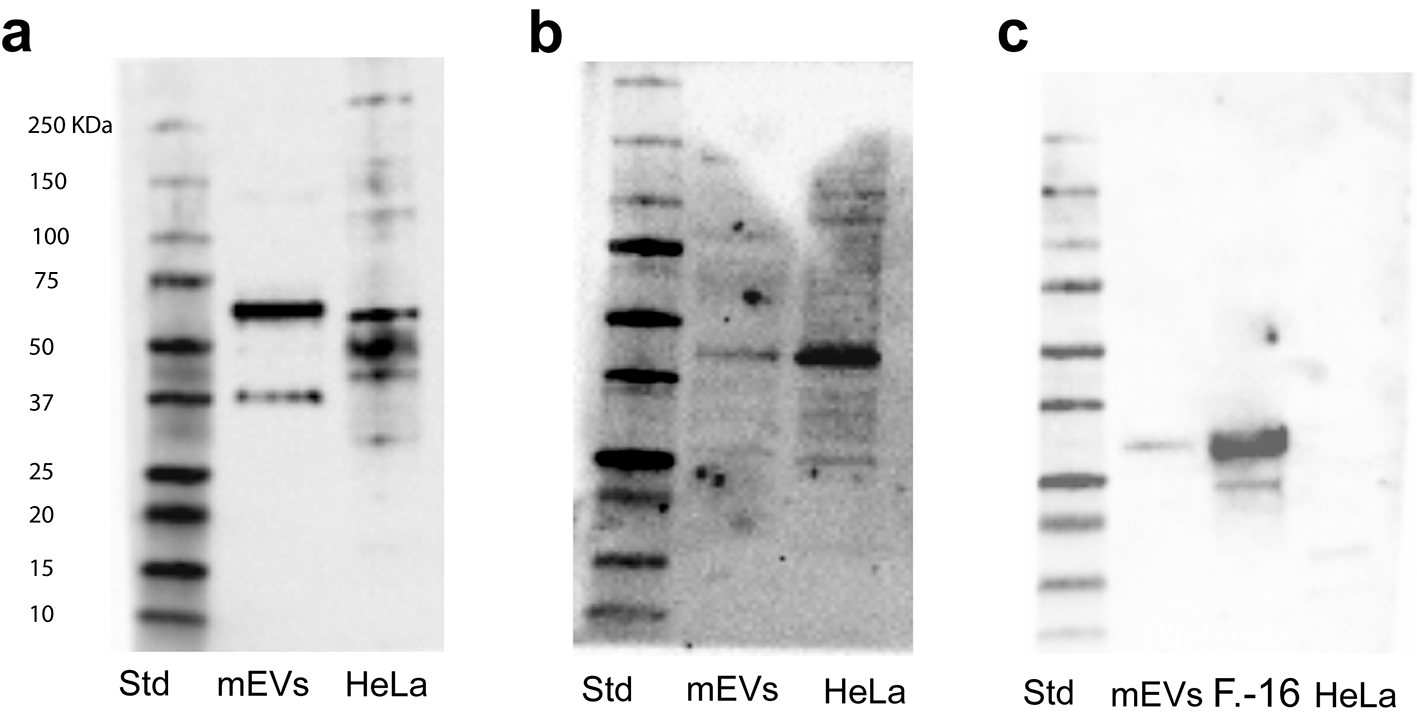

Supplement: Supplementary file 3 — Original, uncropped blot images, including merged molecular weight markers. a) Western blots for Cx43 in mEV lysates and control lysates of HeLa cells with Panx1 CT antibody. b) Western blots for Cx43 in mEV lysates and control lysates of HeLa cells with Panx1 extracellular loop antibody. c) Western blot for casein in the pooled EV fraction, non-EV containing SEC fractions (Fraction 16, F-16) and control lysates of HeLa cells (PNG 252 KB) [file 441_2026_4044_Fig4_ESM.png]

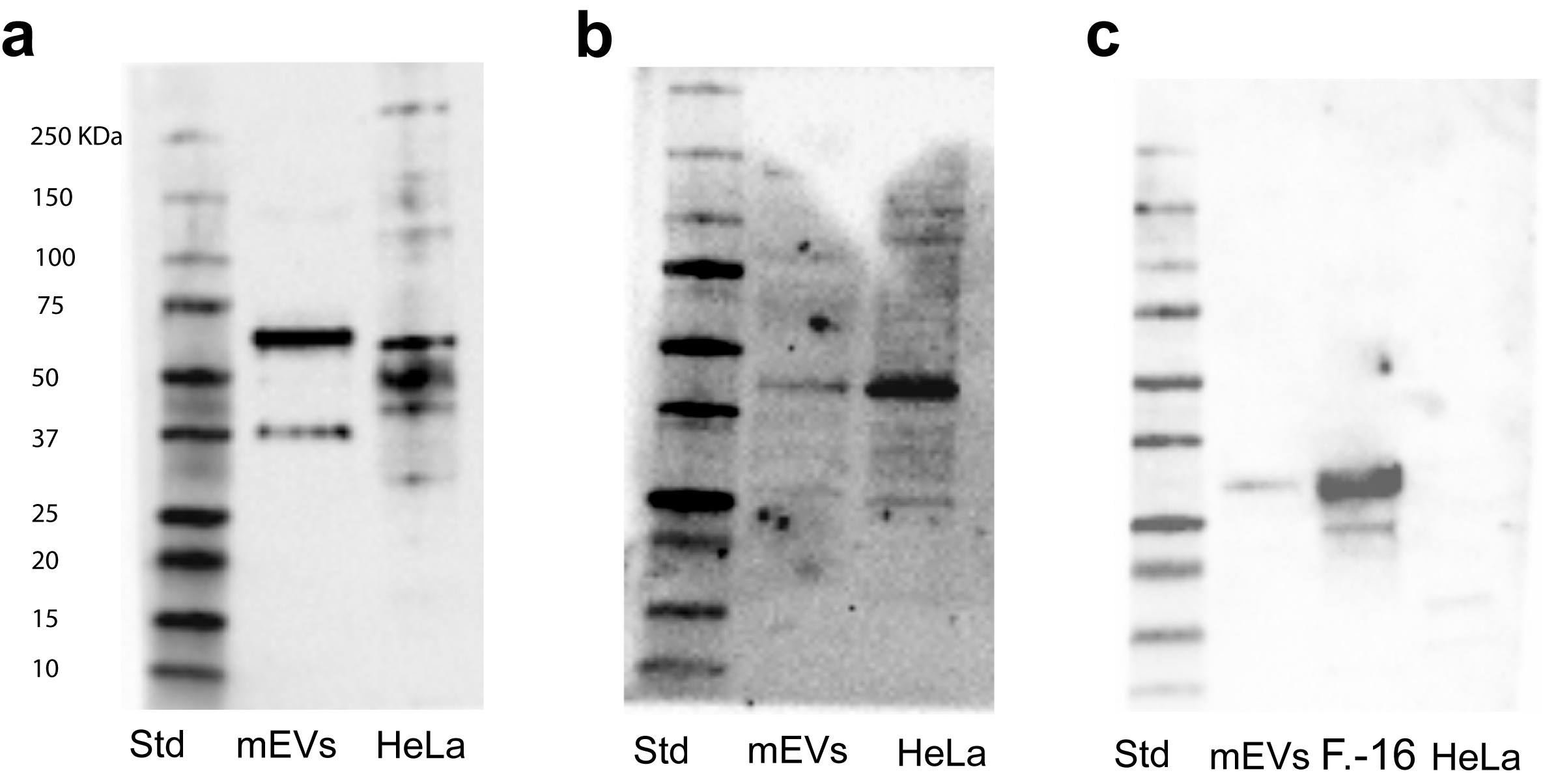

Supplement: Supplementary file 4 — (TIF 11.4 MB) [file 441_2026_4044_MOESM2_ESM.tif]
